# Cellular-Scale Photoreceptor Remodeling After Macular Hole Surgery Examined by Adaptive Optics Optical Coherence Tomography

**DOI:** 10.1167/iovs.67.1.40

**Published:** 2026-01-20

**Authors:** Yuki Hama, Yuki Muraoka, Takahiro Kogo, Naomi Nishigori, Yuki Akiyama, Masaharu Ishikura, Shin Kadomoto, Kenji Ishihara, Akitaka Tsujikawa

**Affiliations:** 1 Department of Ophthalmology and Visual Sciences, Kyoto University Graduate School of Medicine, Kyoto, Japan

**Keywords:** macular hole, retina, retinal imaging, vitreoretinal surgery

## Abstract

**Purpose:**

To investigate outer retinal remodeling at the cellular level after macular hole (MH) surgery using adaptive optics optical coherence tomography (AO-OCT) and to assess the potential clinical relevance of transient hyper-reflective bodies (HRBs) newly observed within the outer nuclear layer (ONL).

**Methods:**

High-resolution AO-OCT was used to longitudinally assess the outer retinal microstructure in 12 eyes from 11 patients with idiopathic MH who underwent successful vitrectomy. The integrity of the photoreceptor inner segment (IS), outer segment (OS), ellipsoid zone, visibility of photoreceptor nuclei in the ONL, and the presence and positional changes of discrete HRBs were evaluated.

**Results:**

All eyes exhibited IS and OS disruptions, focal loss of photoreceptor nuclei, and transient HRBs within the ONL during the early postoperative phase. By the late phase, photoreceptor nuclei reappeared in all eyes, and local restoration of the IS, OS, and ellipsoid zone occurred. The HRB counts decreased over time. The normalized HRB–ELM distances were significantly shorter in the late phase than in the early phase.

**Conclusions:**

This study provides in vivo cellular-resolution evidence for stepwise outer retinal remodeling following MH surgery. Recovery of the photoreceptor segments occurred specifically beneath the reappearing photoreceptor nuclear signals, indicating localized reorganization rather than passive displacement. Transient HRBs diminished in parallel with structural recovery and may represent intermediate stages of retinal remodeling. In conjunction with photoreceptor integrity, HRBs could serve as dynamic indicators of outer retinal repair in vivo.

Retinal repair after macular hole (MH) surgery has been well-characterized at the tissue level using conventional optical coherence tomography (OCT). Postoperative centripetal displacement of the retina^1^^,^^2^ and sequential restoration of the outer retinal structures, including early reformation of the external limiting membrane (ELM) followed by the outer nuclear layer (ONL) and ellipsoid zone (EZ), are recognized contributors to successful MH closure.^3^^–^^5^ These structural changes have been extensively described using conventional OCT. At the cellular level, basic experimental studies have begun to shed light on the underlying reparative mechanisms, and in vitro investigations have demonstrated the regenerative potential of Müller glial cells.^6^^–^^9^ Furthermore, animal models have shown that an inverted internal limiting membrane (ILM) flap can act as a scaffold for Müller cell migration and proliferation, thereby promoting retinal regeneration.^10^ However, despite these insights, the cellular processes underlying photoreceptor and glial responses remain insufficiently understood in the human retina in vivo.

Adaptive optics (AO), originally developed for astronomy,^11^ has recently been integrated into ophthalmic imaging to overcome the limited lateral resolution of conventional OCT.^12^^–^^16^ By correcting higher-order ocular aberrations in real time, AO enables cellular-scale visualization of the retinal microstructures. Using a prototype AO-OCT system, we previously reported fine morphological alterations in healthy eyes and those with epiretinal membranes, including individual photoreceptor nuclei within the ONL, and detailed configurations of the inner segment (IS) and outer segment (OS) of photoreceptors and Müller glia.^17^

In other retinal diseases, discrete hyper-reflective foci (HRF) on OCT have been interpreted as inflammatory cells, lipid deposits, degenerating photoreceptors, or, in the case of AMD, RPE-derived or RPE-phagocytosing cells.^18^^–^^23^ Some studies have also suggested that HRF may reflect transient cellular responses during tissue remodeling.^24^ Whether similar features arise during recovery from MH surgery and whether they reflect aspects of photoreceptor or glial remodeling remains unclear.

To address this gap, we used AO-OCT to examine postoperative remodeling of the outer retina at the cellular level. We focused on the spatial and temporal changes in photoreceptor structures and examined whether any previously undescribed reflective features emerged during retinal repair.

## Methods

### Ethical Approval

The Ethics Committee of the Kyoto University Graduate School of Medicine (Kyoto, Japan) approved this observational study. All protocols adhered to the tenets of the Declaration of Helsinki. All participants provided written informed consent after a detailed explanation of the study objectives, risks, and benefits.

### Participants

AO-OCT images were retrospectively analyzed from eyes that had been imaged as part of an exploratory clinical program assessing the feasibility and potential clinical applications of AO-OCT in various macular diseases. We analyzed eyes that met the following inclusion criteria: (1) patients diagnosed with idiopathic MH who underwent pars plana vitrectomy at Kyoto University Hospital between April 2021 and October 2023 and achieved postoperative hole closure and (2) eyes that underwent AO-OCT imaging at least twice within 1 year postoperatively—once in the early postoperative phase (≤6 months) and once in the late postoperative phase (>6 months). Only eyes that satisfied both criteria were included in the study. The MH diagnosis was confirmed using spectral-domain OCT (Spectralis HRA + OCT; Heidelberg Engineering, Heidelberg, Germany). The exclusion criteria were (1) poor image quality and (2) a history of ocular disease or surgery other than cataract surgery.

All patients underwent comprehensive ophthalmological examinations, including autorefractometry (ARK-530A; NIDEK, Gamagori, Japan), best-corrected visual acuity (BCVA) measurements using a Landolt chart, axial length measurements (IOL Master 700; Carl Zeiss Meditec, Dublin, CA, USA), indirect ophthalmoscopy, slit-lamp biomicroscopy, fundus photography (TRC50LX; Topcon Corp., Tokyo, Japan), and spectral domain OCT.

To illustrate the potential long-term structural outcomes, we included one representative case that underwent AO-OCT imaging 7 years after successful MH surgery. This case was not part of the main cohort or longitudinal analysis.

### Surgical Procedure

All surgeries were performed by a single, experienced vitreoretinal surgeon (YM). Pars plana vitrectomy was performed using a 27G system (Constellation Vision System; Alcon, TX, USA). All eyes underwent either standard ILM peeling or the inverted ILM flap technique after staining the ILM with Brilliant Blue G. All surgeries were performed using SF_6_ gas tamponade. Combined cataract surgeries were performed when necessary.

### AO-OCT Imaging Protocol

We used a prototype AO-OCT device developed in collaboration with Canon Inc. (Tokyo, Japan) capable of simultaneously acquiring AO-OCT and AO scanning laser ophthalmoscopy (AO-SLO) images. The AO-OCT system incorporated a Shack–Hartmann wavefront sensor and a deformable mirror for real-time aberration correction. Aberration correction by the AO system achieved axial and lateral resolutions of 3.4 µm and 3.0 µm, respectively. Images were acquired over a 2.5° (728 µm) field centered on the fovea, with 45 frames per second per B-scan. Retinal tracking using AO-SLO ensured consistent imaging locations across sessions. The captured images were automatically registered and averaged using built-in software. Images were exported at a size of 448 × 1300 pixels (1.126 µm/pixel axially, 1.63 µm/pixel transversely)

### AO-OCT Image Evaluations

The AO-OCT images were assessed for (1) the presence of IS and OS defects, (2) the visibility and relative abundance of individual cone photoreceptor nuclear signals in the ONL, and (3) the presence and spatiotemporal behavior of hyper-reflective bodies (HRBs) within the ONL.

A longitudinal comparison was performed between the early and late postoperative phases within a 400-µm-wide central foveal region. Changes in the apparent distribution of photoreceptor nuclei were qualitatively assessed. Similarly, temporal changes in EZ integrity and in the spatial distribution and appearance of the HRBs were evaluated. Image scaling was corrected for axial length using the modified Littmann formula (Bennett procedure).^25^^,^^26^ All images were pre-processed using a Gaussian filter to reduce noise.

### HRB Definition and Quantification

**Figure 1.**
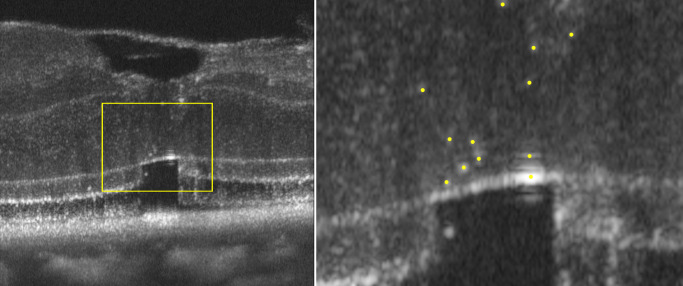
Centroid analysis of HRBs in the foveal ONL. The boxed region (*left*) is binarized. Hyper-reflective bodies are identified using the “Find Maxima” function in ImageJ, and the detected points are overlaid on the original image (*right*) to calculate the centroid positions and compare the positional shifts between the early and late postoperative phases.

HRBs were defined as discrete, ovoid, high-intensity signals within the ONL that exceeded the surrounding nuclei in terms of brightness and size and were not attributable to visible photoreceptor nuclei. To quantify their displacement over time, the AO-OCT images were gamma-corrected and binarized to enhance signal contrast. HRBs were detected using the “Find Maxima” function in ImageJ/Fiji (version 1.54p, National Institutes of Health, Bethesda, MD, USA) (Fig. 1). The total number of detected points was counted, and the detected points were subsequently connected using the “Polygon Selections” tool to form enclosed regions, after which the centroid positions of these regions were identified. These centroid positions were defined as the centroids of the HRBs. In each case, the vertical distance from each centroid to the ELM was measured and normalized to the ONL thickness at the corresponding location to account for variations in retinal thickness and inclination.

Qualitative assessments of the photoreceptor nuclei and ONL structures were initially performed by two independent graders (YH and TK), both of whom were trained in AO-OCT image interpretation. A third senior grader (YM) reviewed all evaluations and resolved any discrepancies to reach a consensus. All image processing was conducted using ImageJ/Fiji software.

### Statistical Analyses

Statistical analyses were performed using JMP Pro version 18 (SAS Institute, Cary, NC, USA). Data are presented as means ± SDs or counts, as appropriate. BCVA was converted to the logMAR for analysis. Differences between the early and late postoperative phases were assessed using the Wilcoxon signed-rank test. Statistical significance was set at a *P* value of less than 0.05.

## Results

Twelve eyes from 11 patients were included in the analysis (Table 1). The mean patient age was 67.8 ± 7.1 years. The mean preoperative BCVA (logMAR) was 0.48 ± 0.22, which improved to 0.16 ± 0.25 at 6 months postoperatively. MHs were classified as stage 2 in eight eyes (66.7%) and stage 3 in four eyes (33.3%). ILM peeling alone was performed in five eyes (41.7%), and the inverted ILM flap technique was used in seven eyes (58.3%).

**Table 1.** Clinical and Surgical Characteristics of the Participants Undergoing MH Surgery

Characteristics	Value
Demographics	
Eyes, n (men/women)	12 (5/7)
Age, years	67.8 ± 7.1
Preoperative parameters	
Axial length, mm	24.6 ± 1.5
Phakic eyes, no. (%)	10 (83.3)
MH stage	
Stage 1, no. (%)	0 (0)
Stage 2, no. (%)	8 (66.7)
Stage 3, no. (%)	4 (33.3)
Stage 4, no. (%)	0 (0)
BCVA, logMAR (Snellen visual range)	0.48 ± 0.22 (20/125–20/25)
Surgical parameters	
ILM peel, no. (%)	5 (41.7)
ILM peel and invert, no. (%)	7 (58.3)
Postoperative parameters	
BCVA (after 6 months), logMAR (Snellen visual range)	0.16 ± 0.25 (20/125–20/16)

**Figure 2.**
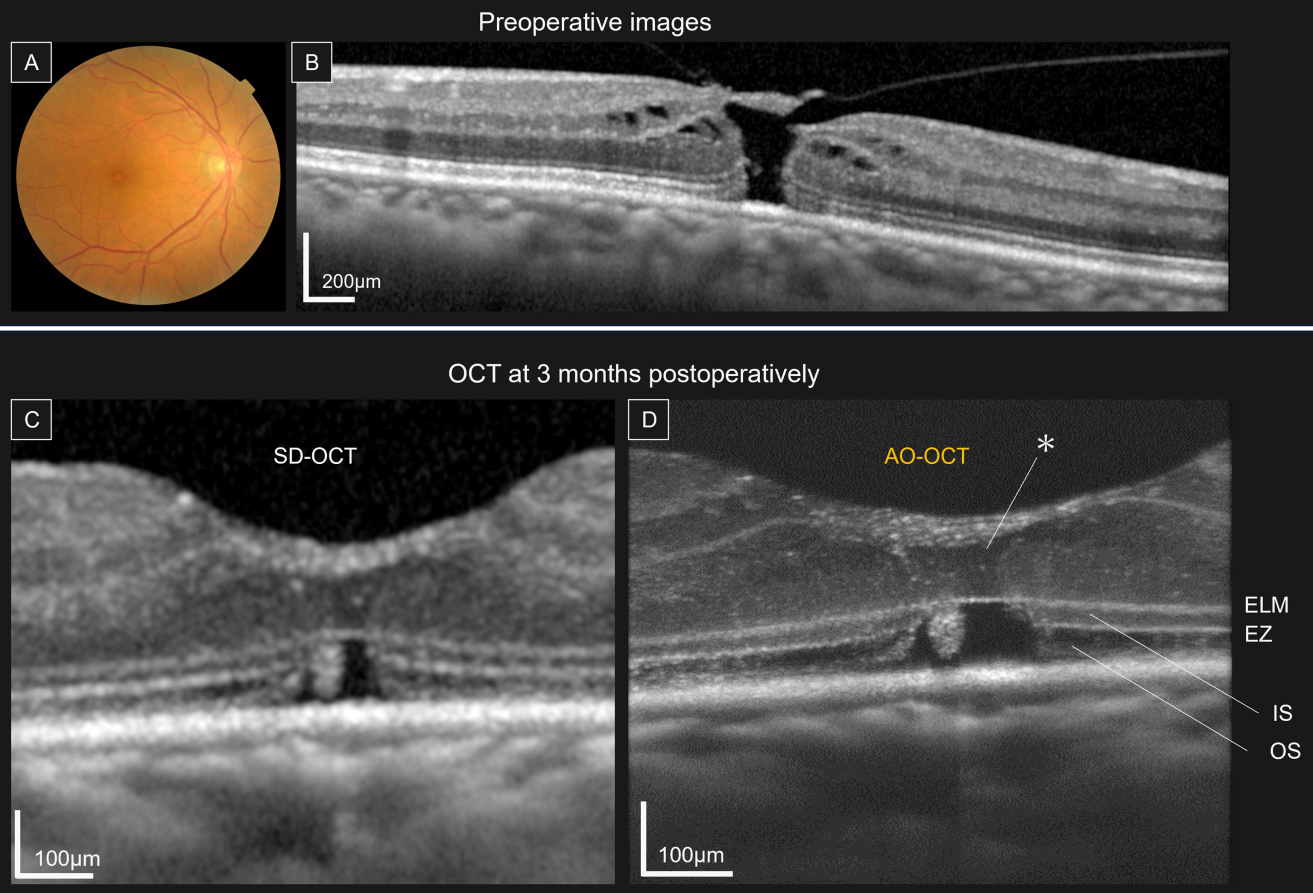
Retinal microstructural changes before and after MH surgery. Representative images illustrating the structural changes before and after MH surgery. (**A**) Preoperative color fundus images. (**B**) Preoperative SD-OCT images showing stage 2 MH. (**C**) Three-month postoperative SD-OCT images showing partial restoration of the outer retinal layers. (**D**) Three-month postoperative AO-OCT images showing IS and OS defects and the absence of photoreceptor nuclei directly above the defects (*). SD-OCT, spectral-domain optical coherence tomography.

In the early postoperative phase (≤6 months), AO-OCT demonstrated consistent structural alterations in all cases. ELM restoration was observed in all 12 eyes. Nevertheless, IS and OS defects were observed in all 12 eyes (100%). Within the ONL overlying the defect area of the IS and OS, two characteristic features were identified in all eyes: (1) focal loss of cone photoreceptor nuclear signals and (2) discrete HRBs that appeared larger and brighter than the surrounding nuclei (Fig. 2).

**Figure 3.**
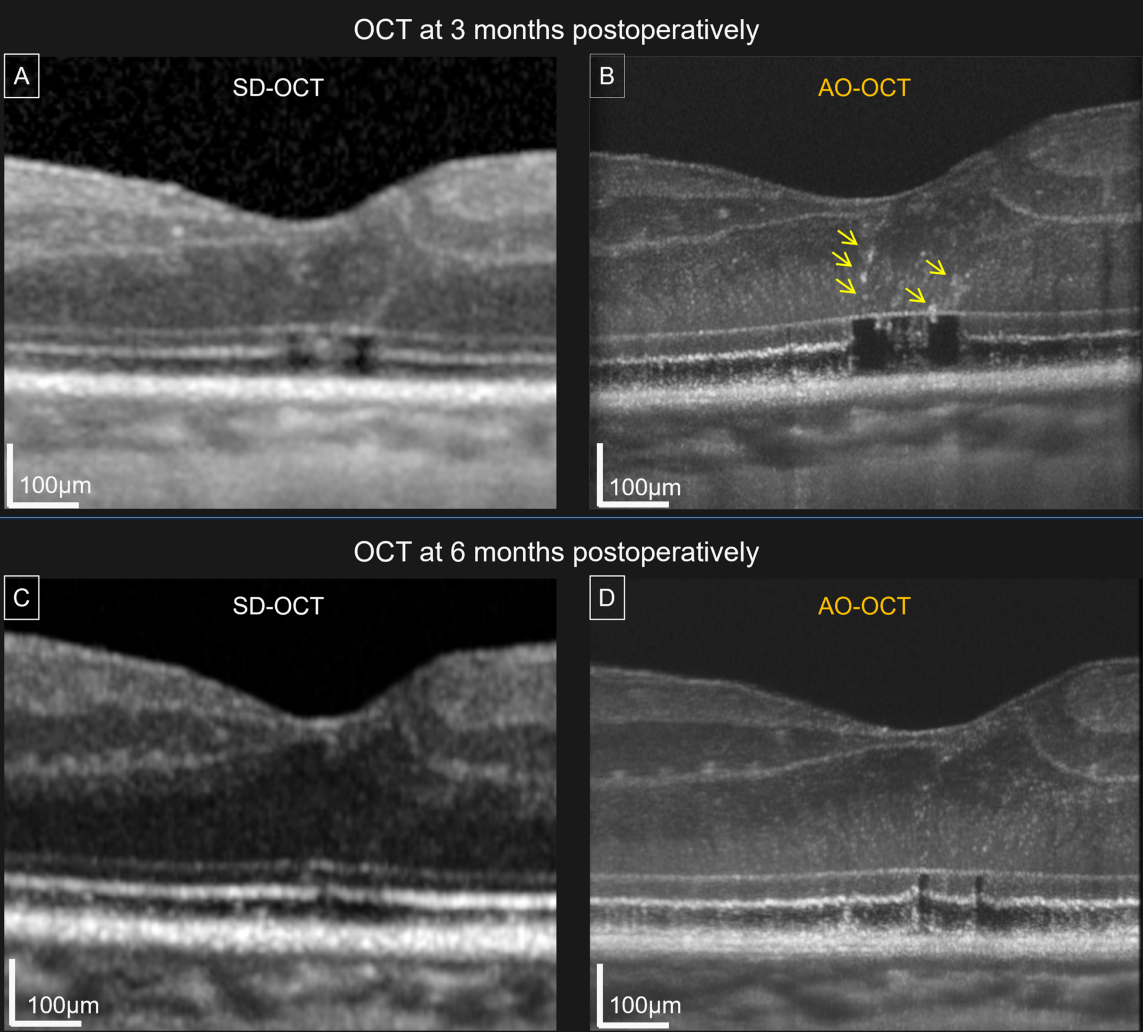
Longitudinal remodeling of the outer retina after MH surgery. (**A**) Three-month postoperative SD-OCT image. (**B**) Three-month postoperative AO-OCT reveals HRBs (*arrows*) located above the disrupted IS and OS, with some extending across the ELM. (**C**) Six-month postoperative SD-OCT image. (**D**) Six-month postoperative AO-OCT reveals decreased numbers of HRBs, IS and OS restoration, and the reappearance of photoreceptor nuclear signals.

A longitudinal analysis comparing the early and late postoperative images revealed reproducible changes in all eyes. In the late phase, photoreceptor nuclear signals became more distinct and widespread within the central 400-µm foveal region in all 12 eyes (100%). Restoration of the IS, OS, and EZ bands was observed in areas corresponding with nuclear reappearance. Quantitative analysis further revealed that the number of HRBs decreased from 4.75 ± 2.6 in the early postoperative phase to 2.08 ± 1.7 in the late phase (*P* = 0.0039, Wilcoxon signed-rank test) (Table 2). Consistently, the number of HRBs decreased over time in all cases (Fig. 3).

**Table 2.** Longitudinal Changes in AO-OCT Findings Between the Early and Late Postoperative Phases

	Postoperative Phase		
Parameters	Early	Late	*P* Value
EZ defect length (µm)	135.84 ± 63.19	58.52 ± 36.21	0.0005^*^
No. of HRBs	4.75 ± 2.6	2.08 ± 1.7	0.0039^*^
ONL thickness (µm)	72.05 ± 12.91	79.95 ± 25.89	0.301
Distance from HRBs centroid^†^ to ELM (µm)	43.78 ± 13.47	34.04 ± 23.45	0.110
Normalized centroid–ELM distance^‡^	0.61 ± 0.16	0.39 ± 0.16	0.0005^*^

* *P* < 0.05 indicates significance. *P* values compare paired measurements between the early and late postoperative phases and are calculated using the Wilcoxon signed-rank test.

† The centroid of each HRB is defined as the geometric center of its binarized signal, as detected by the “Find Maxima” function in ImageJ.

‡ Centroid–ELM distance/ONL thickness.

To assess positional changes in HRBs, gamma correction and binarization were applied to the AO-OCT images, followed by a centroid analysis using ImageJ software. The vertical distance from each HRB centroid to the ELM was measured and normalized to ONL thickness. Although the absolute ONL thickness and centroid–ELM distance did not differ significantly between phases, the normalized centroid–ELM distance was significantly shorter in the late phase compared with the early phase (*P* = 0.0005), indicating a relative downward shift in HRBs within the ONL over time (Table 2).

Additionally, AO-OCT in one representative case imaged 7 years after successful MH surgery demonstrated a persistent absence of photoreceptor nuclei in the ONL, accompanied by IS and OS defects and a lack of EZ reconstruction (Supplementary Fig. S1).

## Discussion

In this study, we used AO-OCT to investigate photoreceptor remodeling at the cellular level after MH surgery, providing insights into microstructural changes that could not be resolved with conventional spectral domain OCT. Early postoperative images revealed focal loss of photoreceptor nuclear signals and disruptions of the inner and outer photoreceptor segments, along with discrete HRBs within the outer retina. Over time, these features progressively resolved in parallel with structural recovery, suggesting that HRBs may represent transient intermediates in the reparative process.

Previous studies have mainly supported the centripetal migration hypothesis,^1^^,^^2^^,^^27^^,^^28^ proposing that residual photoreceptors surrounding the hole migrate toward the foveal center during the repair process. In this study, AO-OCT imaging alone could not determine whether the reappearing photoreceptor nuclear signals within the closed area resulted from cellular migration of existing photoreceptors or from de novo regeneration. However, IS, OS, and EZ reappeared specifically beneath the regions where nuclear signals became visible, suggesting that this process reflects localized structural reorganization associated with the reestablishment of nuclear alignment, rather than simple tissue displacement.

A particularly intriguing aspect is the emergence and temporal dynamics of HRBs within the ONL during the early postoperative phase. These structures progressively shifted toward the ELM and were spatially associated with regions of photoreceptor segment reconstitution. Their behavior suggests that HRBs may reflect active cellular processes during tissue remodeling, including organelle repositioning, cytoplasmic restructuring, or photoreceptor–Müller glia interactions.

Morphologically, HRBs observed in this study resemble the HRF reported in various retinal diseases, including AMD,^22^^,^^23^^,^^29^^,^^30^ diabetic macular edema,^31^^,^^32^ and retinal vein occlusion.^33^ In these conditions, HRF have been associated with worse visual outcomes. In AMD, histological–OCT correlation studies demonstrate that many HRF correspond with transdifferentiated RPE cells or phagocytes containing RPE-specific organelles, indicating ongoing disease activity rather than simple degenerative byproducts.^22^^,^^31^^,^^34^^,^^35^ However, recent findings in rhegmatogenous retinal detachment suggest that early HRF presence may also represent a reparative response.^24^ In eyes with rhegmatogenous retinal detachment, HRF appeared shortly after reattachment, peaked after 3 months, and declined with structural recovery. Early HRF was associated with visual improvement, whereas persistent HRF was correlated with poorer outcomes. These patterns mirror our observations in MH, where HRBs appeared transiently and decreased in parallel with photoreceptor recovery. Collectively, these findings suggest that HRBs may represent temporary structural remodeling during retinal repair, distinct from the persistent HRF seen in chronic degenerative diseases. However, their precise biological nature remains to be clarified.

To further clarify the implications of nuclear loss, we examined a representative case that was imaged 7 years postoperatively. This patient showed a persistent absence of photoreceptor nuclear signals in the ONL. The corresponding IS, OS, and EZ structures failed to recover, even in the long term. These findings suggest that the reappearance of photoreceptor nuclear signals on AO-OCT—consistent with nuclear reorganization—may be necessary for sustained restoration of photoreceptor segments.

Taken together, our findings support a sequential model of outer retinal remodeling after MH surgery, beginning with ELM restoration, followed by the reorganization of photoreceptor nuclei in the ONL and subsequent reconstitution of the IS, OS, and EZ.

Transient HRBs, newly identified using AO-OCT, appeared during the early postoperative period and decreased with structural recovery. Although HRBs share some morphological features with the HRF described on conventional OCT, their transient nature and temporal association with repair suggest that they reflect remodeling-associated, rather than disease-associated, reflective changes. In combination with photoreceptor integrity, HRBs may serve as dynamic indicators of reparative processes in the outer retina. Overall, the present study achieved its primary objective of visualizing postoperative retinal remodeling at the cellular scale using AO-OCT, providing in vivo morphological evidence of dynamic structural recovery not captured by conventional OCT.

### Limitations

This study has some limitations. First, the sample size was relatively small, and the cohort primarily included patients with stage 2 and 3 MH, which may limit the generalizability of the findings to more advanced cases. Second, the study had a retrospective component, because some postoperative AO-OCT images were obtained before formal study initiation, and the inclusion of only eyes with sufficient image quality may have introduced selection bias. Third, surgical techniques varied, with some eyes undergoing the inverted ILM flap procedure and others standard ILM peeling, which may have influenced the remodeling process. Fourth, the timing of postoperative AO-OCT imaging differed among participants. Although AO-SLO–guided imaging ensured consistent targeting, exact image registration across visits was not guaranteed because of the narrow field of view. Finally, the biological identity and functional significance of HRBs remain unclear, because in vivo imaging alone cannot definitively determine their nature. Although AO-OCT provides structural evidence of remodeling, its biological interpretation remains speculative. Future multimodal and histological studies are warranted to elucidate the underlying mechanisms and to clarify the relationship, if any, between HRBs and HRF.

## Conclusions

This study provides in vivo, cellular-scale evidence of spatially coordinated outer retinal remodeling after MH surgery. Photoreceptor segment restoration occurred beneath reappearing ONL nuclear signals, consistent with localized structural reorganization rather than passive displacement. Transient HRBs appeared early within the ONL and decreased with recovery, suggesting an intermediate remodeling phenomenon, although their cellular identity remains unresolved. Prospective studies incorporating histological correlation and preoperative AO-OCT imaging of MHs are needed to test whether HRB-like signals or residual photoreceptor structures predict postoperative restoration and visual outcomes. Although AO-OCT is not yet widely available, its ability to visualize cellular-scale events may refine our interpretation of postoperative changes beyond the limits of conventional OCT.

## Supplementary Material

Supplement 1
