# Supplementary material for: Cellular-Scale Photoreceptor Remodeling After Macular Hole Surgery Examined by Adaptive Optics Optical Coherence Tomography
Source: Invest Ophthalmol Vis Sci. 2026 Jan 20;67(1):40. doi: 10.1167/iovs.67.1.40 (PMC12831147; doi:10.1167/iovs.67.1.40)
Supplement: Supplement 1 [file iovs-67-1-40_s001.pdf]

Title: Cellular-Scale Photoreceptor Remodeling After Macular Hole Surgery Examined by Adaptive Optics Optical Coherence Tomography

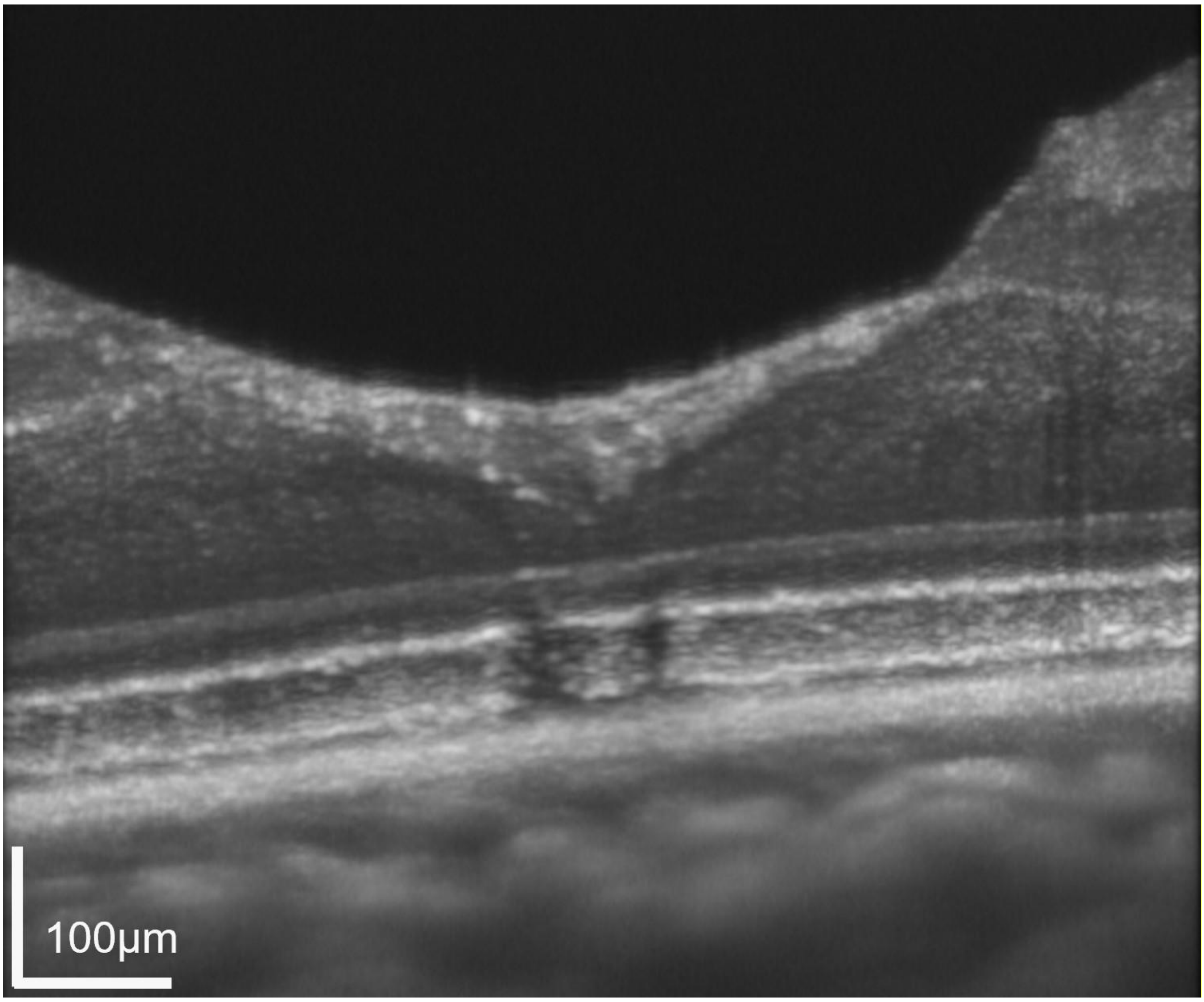

Supplementary Figure 1. Persistent photoreceptor defects seven years after macular hold surgery.

Adaptive optics optical coherence tomography images acquired seven years postoperatively from an eye with a previously closed macular hold show the persistent absence of photoreceptor nuclei in the outer nuclear layer. Corresponding defects in the IS and OS and a lack of EZ reconstruction are also visible. These findings suggest an association between the failed repopulation of photoreceptor nuclei and the long-term absence of IS, OS, and EZ.

EZ, ellipsoid zone; IS, inner segment; OS, outer segment.
